# Approaches in Immunotherapy, Regenerative Medicine, and Bioengineering for Type 1 Diabetes

**DOI:** 10.3389/fimmu.2018.01354

**Published:** 2018-06-12

**Authors:** Christopher Kopan, Tori Tucker, Michael Alexander, M. Rezaa Mohammadi, Egest J. Pone, Jonathan Robert Todd Lakey

**Affiliations:** ^1^Department of Surgery, University of California Irvine, Irvine, CA, United States; ^2^Department of Cell and Molecular Biosciences, University of California Irvine, Irvine, CA, United States; ^3^Department of Chemical Engineering and Materials Science, University of California Irvine, Irvine, CA, United States; ^4^Department of Pharmaceutical Sciences, University of California Irvine, Irvine, CA, United States; ^5^Department of Biomedical Engineering, University of California Irvine, Irvine, CA, United States

**Keywords:** type 1 diabetes, autoimmunity, regulatory T cell, immunosuppression, transplantation immunology

## Abstract

Recent advances on using immune and stem cells as two-pronged approaches for type 1 diabetes mellitus (T1DM) treatment show promise for advancement into clinical practice. As T1DM is thought to arise from autoimmune attack destroying pancreatic β-cells, increasing treatments that use biologics and cells to manipulate the immune system are achieving better results in pre-clinical and clinical studies. Increasingly, focus has shifted from small molecule drugs that suppress the immune system nonspecifically to more complex biologics that show enhanced efficacy due to their selectivity for specific types of immune cells. Approaches that seek to inhibit only autoreactive effector T cells or enhance the suppressive regulatory T cell subset are showing remarkable promise. These modern immune interventions are also enabling the transplantation of pancreatic islets or β-like cells derived from stem cells. While complete immune tolerance and body acceptance of grafted islets and cells is still challenging, bioengineering approaches that shield the implanted cells are also advancing. Integrating immunotherapy, stem cell-mediated β-cell or islet production and bioengineering to interface with the patient is expected to lead to a durable cure or pave the way for a clinical solution for T1DM.

## Introduction

Diabetes mellitus is a disease of endocrine pancreas with the main symptoms being high blood glucose levels or hyperglycemia. This hyperglycemic state results in lack of glucose in tissues and organs, leading to their impairment, which, if untreated, can lead to tissue pathology, necrosis, and ultimately death. There are two primary categories of the disease: type 1 diabetes mellitus (T1DM) and type 2 diabetes mellitus (T2DM). T1DM is understood to be mostly of an autoimmune etiology and often onsets prior to age 20–30, which is why T1DM is also referred to as juvenile-onset diabetes (1, 2). T2DM on the other hand is a chronic metabolic disorder understood to be caused by various genetic components and insulin insensitivity (3), i.e., insufficient insulin–insulin receptor signaling in tissues and, therefore, insufficient glucose uptake from the bloodstream and into cells. Variety of potential mechanisms have been proposed to be responsible for pathogenesis of T2DM, including glucotoxicity, lipotoxicity, oxidative stress, endoplasmic reticulum stress, and amyloid deposition. This category of the disease has recently been divided into three subtypes: subtype 1 expresses increased risk of developing neuropathy and retinopathy, subtype 2 with increased risk of cancer malignancy and various cardiovascular diseases, and subtype 3 with increased risk of neurological and cardiovascular disease along with increased risk of allergies and HIV (4). As such, these subtypes require different treatments depending on the classification, i.e., drugs for blood pressure control vs treatment with exogenous insulin. Nevertheless, a fraction of T2DM patients may have some destruction of pancreatic β-cells and, therefore, also classify as T1DM patients (2).

Recently, there have been advances in the areas of islet transplantation and immunotherapy to restore the body’s immune tolerance to the pancreas, and stem cell therapy as possible long-term cellular treatments for T1DM (5–7). These methods show promise as treatment for T1DM as they work to halt the chronic autoimmune attacks against any remaining or newly produced endogenous β-cells as well as transplanted insulin-producing cells from stem cells or pancreatic islets from organ donors. Although treatment for both T1DM and T2DM is needed, in here we will focus on recent advances in immunological and stem cell treatments as a two-pronged approach for T1DM treatment in addition to biotechnological treatments.

## Immune Etiology of T1DM

**Figure 1:**
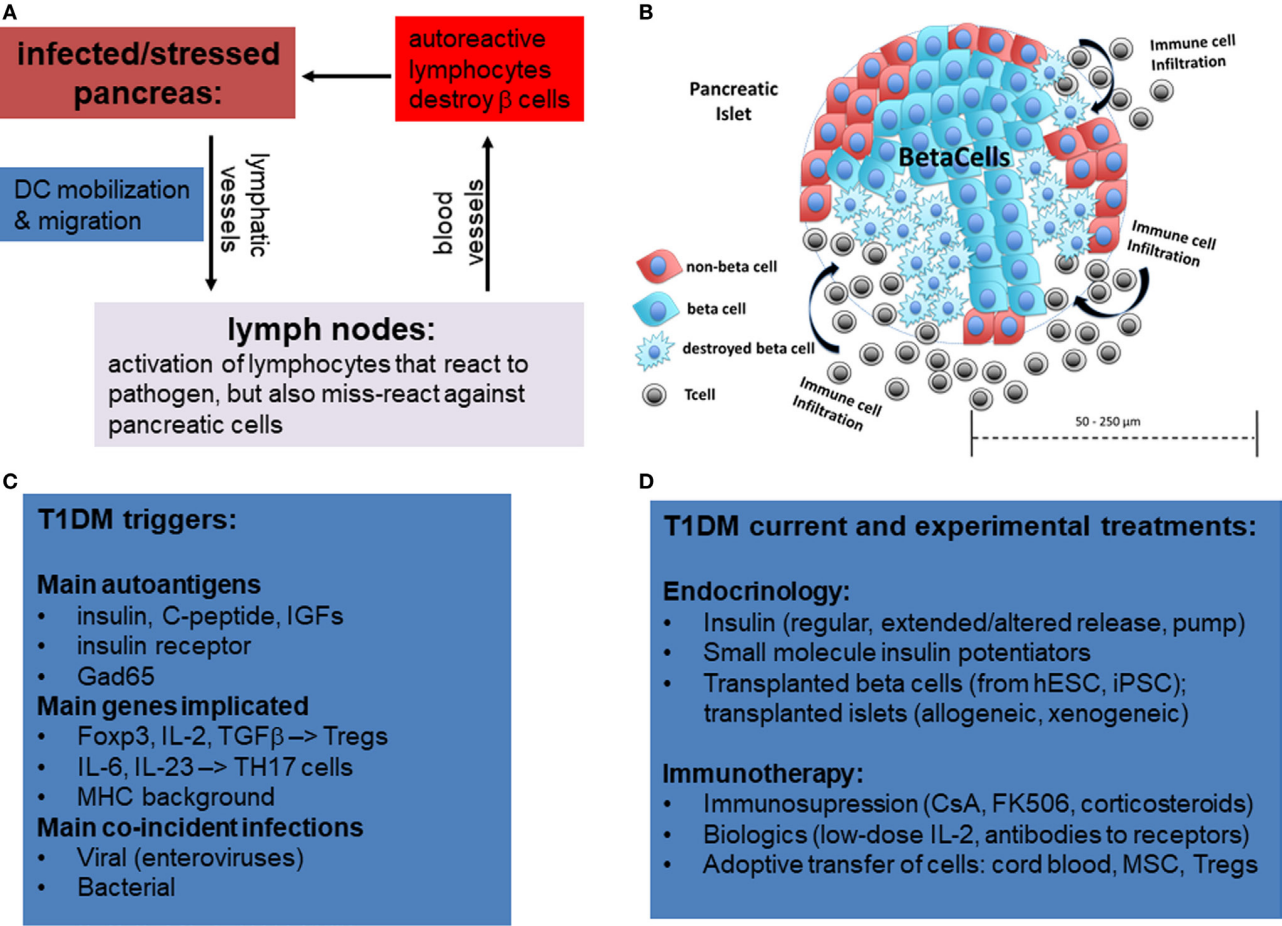
Overview of mechanisms of etiology of type 1 diabetes by miss-guided autoreactive adaptive immune response following infection, major stress, or trauma to the pancreas. **(A)** Following bacterial or viral infections or trauma in the pancreas, dendritic cells (DCs) are activated. While DCs normally only present on their MHCI and MHCII antigens from pathogens, in rare circumstances DCs also activate T lymphocytes that have some affinity for autoantigens, a phenomenon referred to as “molecular mimicry.” Acute destruction of endocrine pancreatic cells, especially β-cells, can then give rise to type 1 diabetes mellitus (T1DM). **(B)** Representative diagram showing infiltration of pancreatic islets by immune cells and partial islet destruction. **(C)** The main postulated triggers of T1DM include autoantigens, certain mutations in genes activating regulatory T-cells or Th17 cells, and viral and bacterial infections. **(D)** Overview of current and experimental approaches using immune and stem cells to treat T1DM.

Type 1 diabetes mellitus is thought to be of an autoimmune etiology (Figure 1A). However, the specific mechanisms by which the immune system attacks the pancreas are not yet entirely clear. It is generally understood that the body becomes self-reactive to various autoantigens in the pancreas that can produce insulitis (Figure 1B), an inflammatory response in the Islets of Langerhans, the smallest autonomous cell clusters of endocrine pancreas consisting of α cells (producing glucagon) β-cells (producing insulin), γ cells (producing pancreatic polypeptide), δ cells (producing somatostatin), and ε cells (producing ghrelin). The collective action of these cells, which is in large part due to their secretion of respective peptide hormones, maintains the physiological islet structure and function (1–3).

Although the causes may vary for each patient or patient strata, there is evidence that β-cell loss in T1DM can occur following acute infectious disease (8, 9) or sterile trauma (10), which can be further exacerbated by genetic disposition (Figure 1C). There are two primary reasons in which these mechanisms are suspected to cause the disease. First, following infection or trauma the immune system is in a heightened activation state and, therefore, there are a greater number of primed lymphocytes that may cross-react to self-antigen. Second, there is a higher concentration of cytokines as well as adhesion and co-stimulatory molecules on antigen-presenting cells (APCs) and parenchymal cells which can sustain the activation of primed cross-reactive lymphocytes.

**Figure 2:**
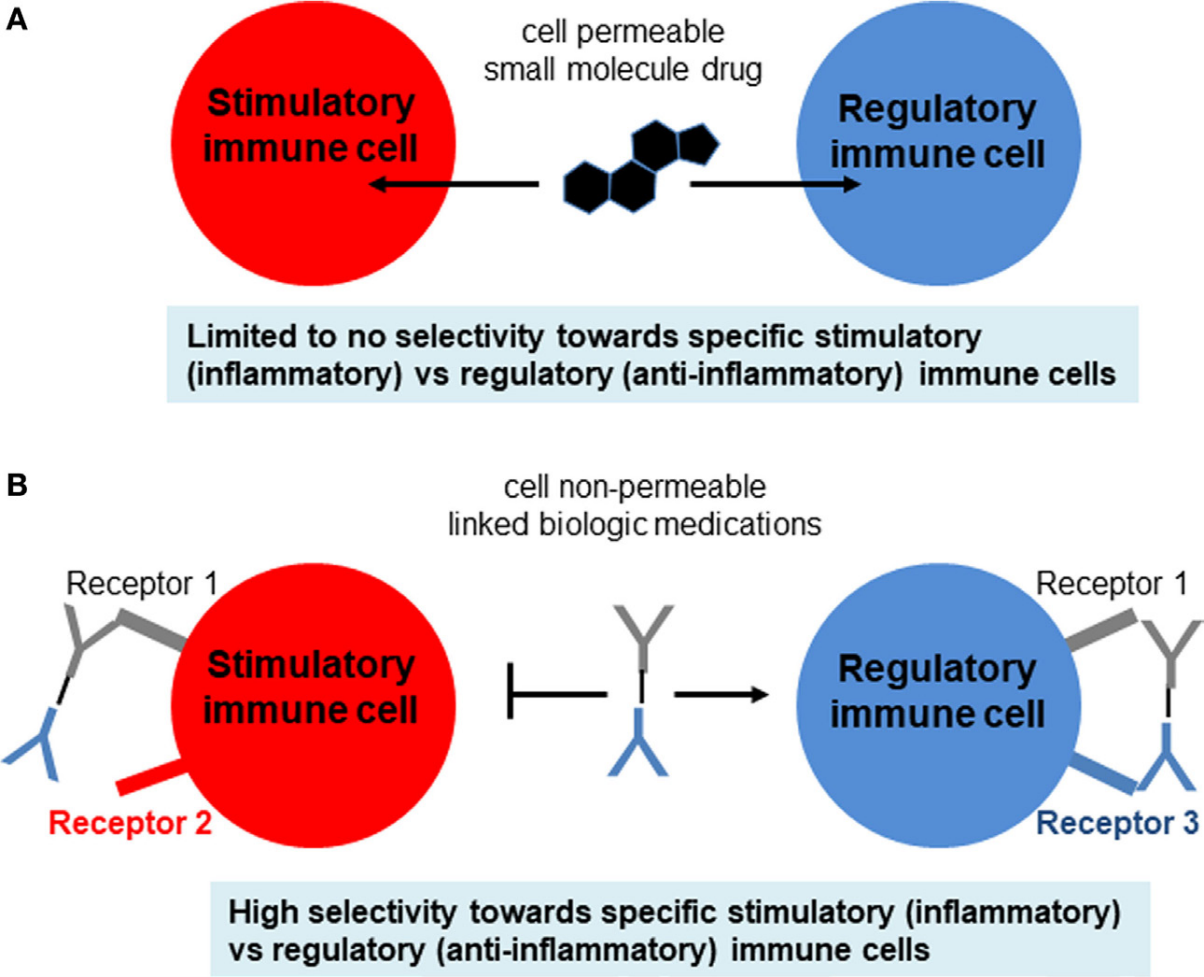
Comparison of small molecule drugs and complex biologics. **(A)** Small molecule drugs for immunosuppressive therapy include the well-known corticosteroids, nucleotide synthesis inhibitors, NFAT pathway inhibitors, and mTOR pathway inhibitors. Although some immune cells may express more intracellular targets than other types of immune cells, typically the selectivity of small molecule drugs for target vs off-target cells is not high enough and, therefore, leads to side effects. **(B)** Biologics include antibodies (which can be blocking, stimulatory, or inhibitory), cytokines, and cytokine receptors or decoys that either amplify or attenuate cytokine signaling. These biologics are usually administered as pure agents, or are further integrated on carrier platforms (such as nanoparticles, liposomes, polymers). If biologics are linked to each other or to carriers/platforms, then additional considerations apply to optimization of linkers (free, fused, tagged, covalent).

Currently, the general treatment for T1DM is close monitoring of daily blood glucose levels with injections of long-acting insulin (e.g., Lantus, Levemir) and short-acting/mealtime insulin (e.g., Humalog, Novolog). Meanwhile, the hyperglycemia associated with T2DM is often treated with various forms of insulin as well as small molecule drugs that either increase insulin sensitivity (e.g., metformin) or assist with the excretion of excess sugars (e.g., canagliflozin) (Figure 1D). However, these treatments for both diseases typically involve small molecule drugs with limited selectivity toward target cells (Figure 2A), and, therefore, can be viewed as temporary solutions, with patients typically continuing these respective treatments indefinitely after onset.

### Clinical Onset and Development

Clinically, T1DM presents as sudden, unexplained glucose elevation, with symptoms of fatigue, polyuria, polydipsia, and headaches (1, 2). T1DM afflicts predominantly individuals prior to age 20, leading to the other term of juvenile diabetes. The connection between childhood and self-reactive immune attack has been proposed to arise, in part, from the misdirected attack of the immune system against new self-antigens that are expressed rapidly and that the immune system fails to recognize as (maturing) self. Likewise, in the case of T1DM following viral or bacterial illness, it is thought that lymphocytes may react to self-antigens that are very similar to the pathogenic antigens (11) (Figure 1C). Although not identical to foreign antigen, the epitopes of a self-antigen may nevertheless bind sufficiently to primed/activated T cells, with concomitant signaling from bystander cytokines and co-stimulatory/adhesion receptors. This kind of activation is known as molecular mimicry and is seen in other autoimmune diseases, such as rheumatoid arthritis and multiple sclerosis (12, 13).

### Immune Involvement in Disease Onset and Progression

**Figure 3:**
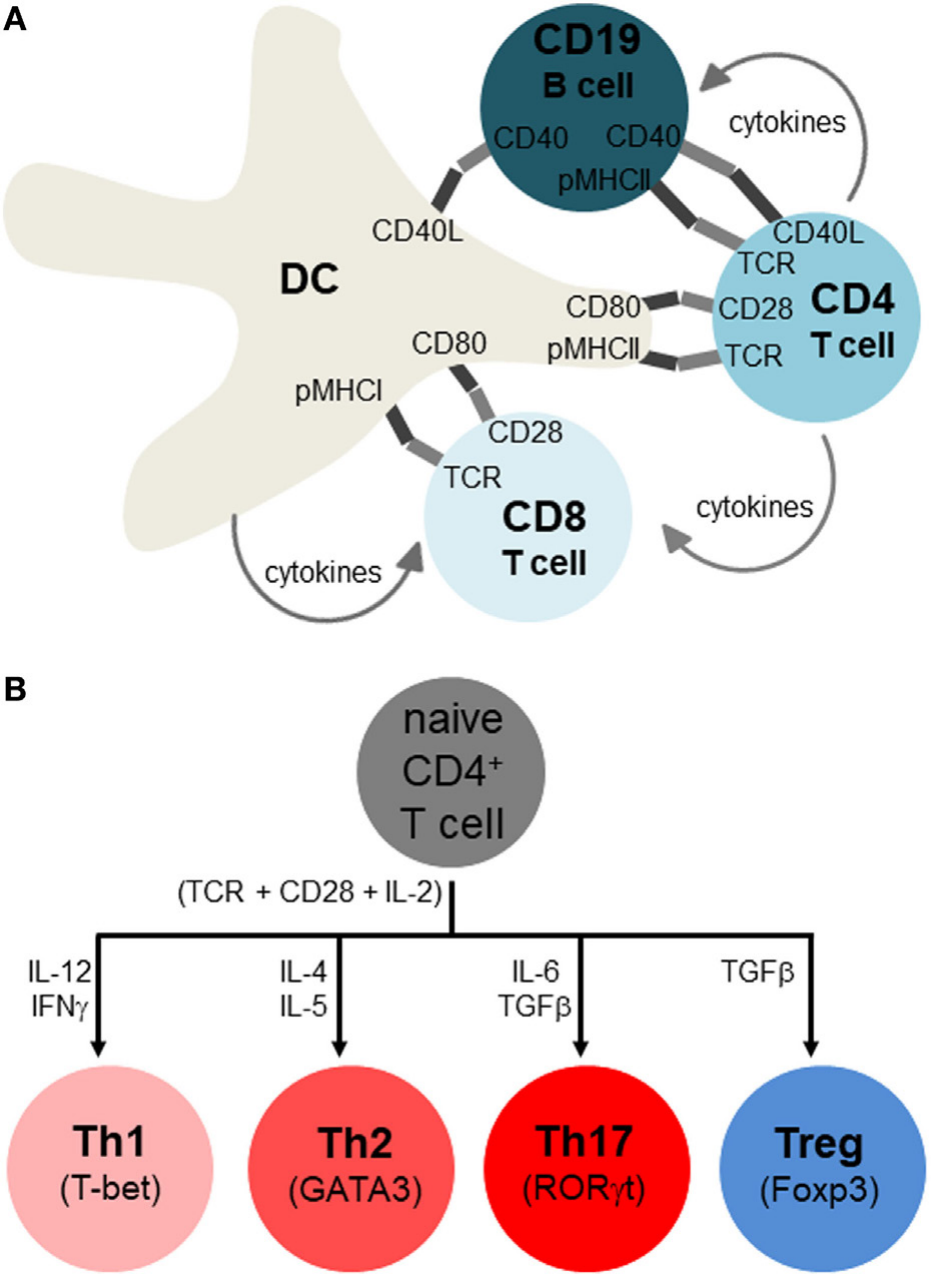
Adaptive immune response and its polarization for different classes of infection or stress. **(A)** Antigen-presenting cells include dendritic cells (DCs), macrophages, and B cells, but only DCs are thought to initiate activation of naïve T lymphocytes, with macrophages and B cells sustaining activation of already primed T cells. These immune cells interact by both membrane-spanning protein receptors displayed on the cell surface (dark and light segments), as well as soluble growth factors and cytokines. The main activating signals have been traditionally referred to as signal 1 (antigen specificity), signal 2 (co-stimulation), signal 3 (growth factor), and signal 4 (differentiation, polarization to distinct cell types). Note that not all of these interactions occur simultaneously, and that while this depiction occurs in secondary organs (lymph nodes, spleen), similar interactions also occur in peripheral tissues, especially near sites of infection or pathology where APC-lymphocyte clusters may assemble to ectopic lymphoid clusters. **(B)** Overview of T helper cell differentiation. Antigen specificity (signal 1), co-stimulation (signal 2), and growth factor (signal 3) are postulated to activate CD4 T cells (indicated right below the naïve CD4 T cells), with signal 4 (polarizing cytokines) skewing them to particular Th subsets (whose identity is maintained by the master transcription factors shown).

T and B lymphocytes sense antigens differently, with B cells binding antigen directly and T cells only binding antigens that is presented on MHCI (for CD8 T cells) and MHCII (for CD4 T cells) complexes on the membranes of APCs. The most potent APCs are dendritic cells (DCs), which can activate naïve T cells, whereas macrophages and B cells are weaker APCs that sustain the activation and differentiation of already primed T cells (14) (Figure 3A). Additionally, non-hematopoietic cells in the periphery may express MHCII under various stressful conditions and may further influence lymphocyte differentiation (15). The receptor clusters that sense antigens are referred to as B cell receptors (BCRs) and T cell receptors (TCRs). BCR and TCR signaling are referred to as immune signal 1, indicating the primary and indispensable role of this signal in eliciting lymphocyte activation. However, for full and proper lymphocyte activation and differentiation, signal 1 must be combined with co-stimulatory signal 2 (e.g., CD28, TNFRs, TLRs), and growth and differentiation signal 3 (e.g., IL-2 for T cells and IL-4 for B cells). Finally, signal 4 is referred to as “polarization,” and is shaped up by a myriad of cytokines and other factors as will be detailed below (Figure 3B).

Thus, besides engaging self-antigen by mistake *via* TCRs or BCRs, T and B lymphocytes also require additional signals from proteins on the cell membrane as well as soluble factors before engaging in an autoimmune attack. The main co-stimulatory protein in T cells is CD28, whereas after the first T lymphocyte division IL-2 sustains the lymphocyte clonal expansion (16). CD28 is engaged by receptors of the B7 family, namely CD80 and CD86. A second B7 ligand, CD152 (CTLA-4) is thought to also bind to CD80 and CD86 at even higher affinity than CD28 thereby downregulating the T cell activation (17). However, since CTLA-4 is found at high levels on regulatory T-cells (Tregs), it may also exert its observed immunosuppressive role *via* Tregs without affecting the initial TCR-CD28-mediated priming and activation. Many other modulatory receptors, either stimulatory or inhibitory for particular cell types and conditions have been found, especially those of the TNFR superfamily like CD134, CD137, and CD357 (18, 19). These receptors may be more active in the peripheral tissues, and may differentially modulate T cell subtypes, such as CD4, CD8, and Tregs.

The fourth polarization signal determines which subtype immune cells become after initial priming, such as CD4, Th1 (IFNγ), Th2 (IL-4), Th17 (IL-6, TGFβ1), and Tregs (IL-2, TGFβ1) (Figure 3B). There is a similar, though not identical, cytokine direction profile for cytotoxic CD8, though they are generally divided into TC1 (IFNγ), TC2 (IL-4), and Tregs (20–24) (Figure 2B). T helper cell differentiation of naive unpolarized Th0 cells requires concomitant engagement of the TCR, co-stimulatory receptors of the B7 and TNFR family, as well as the polarizing cytokine(s) (21–27). CD4 T helper cells provide “help” to CD8 cells mostly in the form of soluble cytokines, such as IL-2, although receptor–ligand interactions are also involved (28, 29) (Figure 2A). The subpopulations referred to as Th0, Th1, Th2, Th17, and Treg are important because the type of response can influence the overall pathology and inflammation. Th2 conditions activate/skew monocytes/macrophages toward the M2 alternatively activated phenotype, which is less destructive than M1. Th17 is the most destructive subset, since IL-17 fuels cytotoxic CD8 T cells (25). Another critical cytokine is interleukin-21 (IL-21) which has an important role in maintenance and function of both T cells and B cells. The receptor for IL-21 is distributed on lymphohematopoietic cells and IL-21 is predominantly produced by activated CD4 T cells and natural killer cells. The principle role of IL-21 is promotion of B cell activation, differentiation, or death during humoral immune responses. Increased production of IL-21 can lead to autoimmune disease and enhanced autoantibody production. IL-21 is capable of inhibiting TGFβ for the expression of Foxp3 by T cells which leads to a switch in the differentiation pathway away from Tregs and toward Th17 cells (30). IL-21 blockade leads to a reduction in immune cells infiltration into the islets, and CD8 T cells mediated islet graft rejection was found to be IL-21 dependent (31). *Il21r*^−/−^ non-obese diabetic (NOD) mice have shown to be protected from T1DM and fail to develop insulitis (32).

Regulatory T-cells are found in both CD4 and CD8 T cell lineages, and further express the high affinity IL-2 receptor α chain (CD25) and the master transcription factor Foxp3. However, the CD4 Tregs have been more thoroughly characterized as they are more abundant *in vivo*. Interestingly, CD4 Tregs may be subdivided into Th1-Tregs, Th2-Tregs, and Th17-Tregs, with recent evidence hinting that each of these Treg subsets suppresses its own respective-activated subset (33) more than other subsets. Another more radical view emphasizes the role of signal combinations that result in a continuum of T cell phenotypes (34) over that of distinct and unchangeable T cell subsets. Regardless of the actual stability and plasticity of T cell subsets, the role that extracellular signals emanating from pathogens as well as from immune cells (mainly in the form of cytokines) is widely agreed upon to be a strong determinant of the ensuing immune responses (35, 36) Importantly, the form of presentation of the signals (whether soluble, membrane-bound, or inter-synaptic), and their concentration, timing, and build-up (hysteresis) (37) are expected to play key roles in determining T cell polarization (38, 39).

The Th17 helper T cell reaction is particularly destructive to tissues (40) and has been implicated as a leading cause of β-cell destruction in T1DM. Th17 cells are a specialized subset of CD4 T cells that produce the proinflammatory cytokine IL-17, and these cells are expanded in the pancreatic-draining lymph nodes (PLN) of T1DM patients relative to healthy subjects (41). IL-17 was produced in the PLNs of diabetic subjects but not healthy subjects when the PLN was exposed to diabetes specific antigens such as GAD65 or pro-insulin. This differs from the production of IFNγ, which was found to be similar between the two groups. Treatment of 10 weeks old NOD mice with anti-IL-17 antibody decreased the amount of T cell islet infiltrates. Furthermore, it was noted that the treatment with anti-IL-17 antibody decreased GAD65 autoantibody levels (42). Interestingly, treatment with IL-25 was more effective in reducing the production of GAD65 autoantibody levels relative to anti-IL-17 treatment in addition to restoring euglycemia after islet transplant. Paradoxically, treatment with anti-IL-17 increased the number of GAD65 reactive splenocytes, while IL-25 decreased the number of autoreactive Th17 T cells. Another interesting observation was that the expression of CD4 Tregs was decreased in the PLN (41). Tregs function as modulators of the immune system by suppressing the action of autoreactive T cells and, therefore, hold great promise in the treatment of autoimmune diseases (43).

## Immunotherapy Using Biologics and Cells

**Figure 4:**
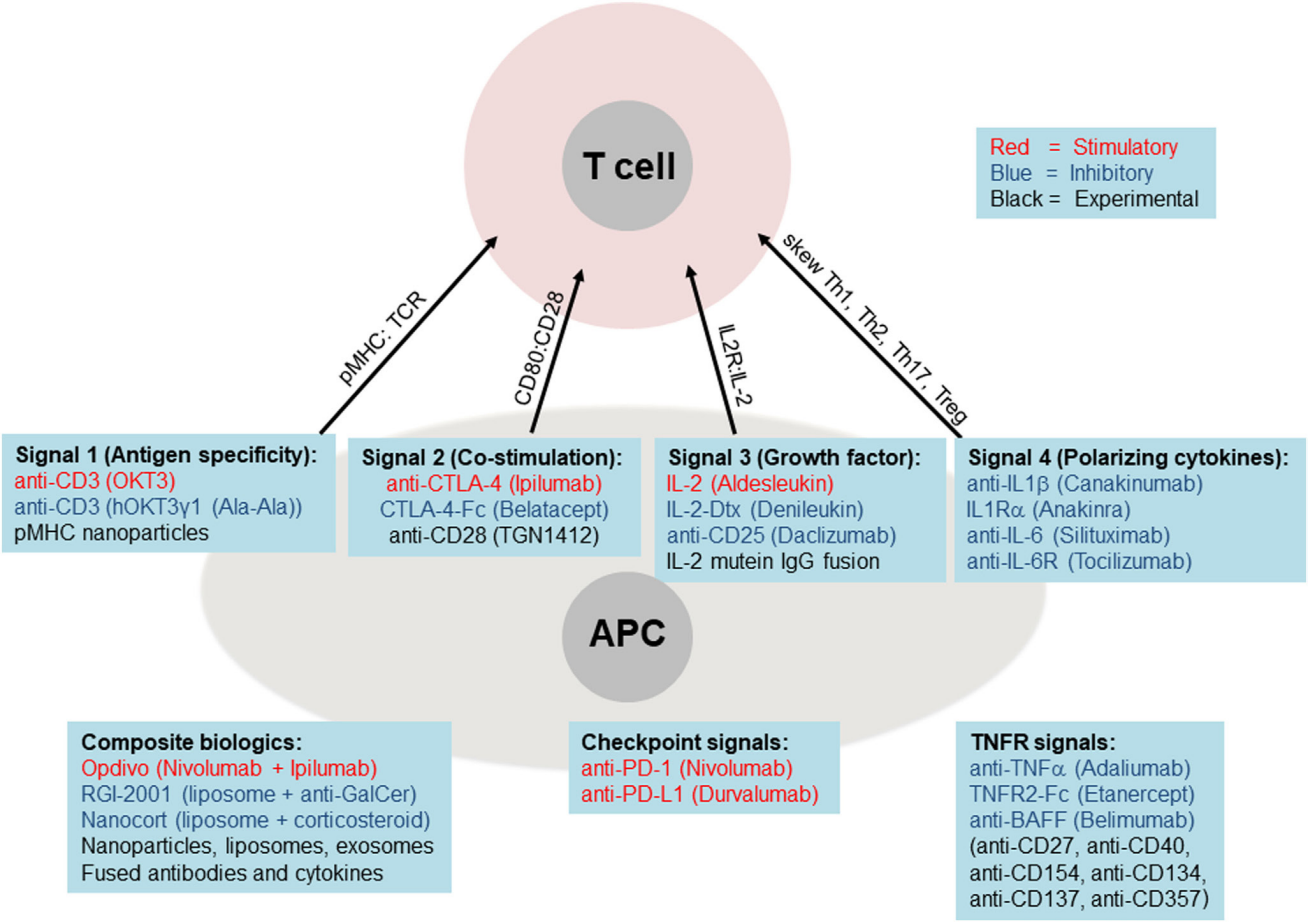
Biologics targeting core immune pathways. Current (FDA-approved) biologic medications are shown as stimulatory (red font), inhibitory (blue font), or experimental (black font). These biologics are listed below each of the main four pathways, plus the critical TNF receptor and checkpoint pathways. The lower left panel lists examples of composite biologics composed of two or more biologics and carriers.

With the advent of new immunological treatments and technology, immunotherapy holds promise as a possible treatment for autoimmune diseases like T1DM (44–46) (Figure 4). Several methods of selective expansion of tolerance-inducing immune cells, such as Tregs, tolerogenic DCs, and M2 macrophages, are currently under consideration, typically using protocols based on stimulations by biological macromolecules. The shift from the traditional small molecule drugs to larger macromolecules either synthesized chemically or produced and purified from cells (referred to as biologics) was necessitated in large part due to the limited selectivity of small molecules for specific immune cell subsets (Figure 2A). This ultimately resulted in their activity in non-target cells and, therefore, to loss in efficacy compounded by severe side effects. Small molecule drugs for immunosuppressive therapy include the well-known corticosteroids, nucleotide synthesis inhibitors, NFAT pathway inhibitors, and mTOR pathway inhibitors. Although some immune cells may express more intracellular targets than other types of immune cells, typically the selectivity of small molecule drugs for target vs off-target cells is not high enough and, therefore, leads to side effects.

By contrast, a newer class of medications is being built around biologics, which include antibodies (blocking, stimulatory, or inhibitory), cytokines, and cytokine receptors or decoys that either amplify or attenuate cytokine signaling (Figures 2A and 4). These biologics are usually administered as pure agents, or are further integrated on carrier platforms (such as nanoparticles, liposomes, and polymers (47)). If biologics are linked to each other or to carriers/platforms, then additional considerations apply to optimization of linkers (free, fused, tagged, and covalent). The end result is typically a medication that has a high capacity to target specific immune cell subsets without stimulating unintended cell targets. These medications can be administered by healthcare professionals or self-administered by the patient, at frequencies from weekly to once every several months. Issues of costs, reliability, and quality of biologics are also improving. More importantly, the potential capability to induce long-term tolerance in the patient, whether by selective manipulation of tolDCs, M2 macrophages, or Tregs, raises the possibility of selective immune tolerance toward transplantation of the pancreatic organ, islets of Langerhans, β-cells, or various differentiated pancreatic stem and progenitor cells. The ultimate goal would be to combine the emerging immunotherapy approaches based on cells and biologics with bioengineering approaches using biocompatible materials and stem cells as a dual approach for a permanent cure for T1DM.

### Targeting the Antigen Specificity “Signal 1” and Co-Stimulatory “Signal 2”

T cell receptor (TCR) signaling is initiated after the TCR recognizes peptides presented by cognate antigen-presenting cells (APCs, mainly DCs, macrophages, and B cells). In T1DM, some of the main candidate self-antigens include GAD65, insulin (especially in its unique forms found only in the pancreas, namely full length unprocessed pro-insulin, C-peptide, as well as the zinc-coordinated hexameric crystals of mature insulin), glucagon, and IGF-1 and its receptors (11, 48) (Figure 1C). The candidate antigens are fairly unique to pancreatic cells and tissues as opposed to being ubiquitously expressed in the body. Correlations between T1DM and other autoimmune diseases has been weak, but there is genetic evidence that genes of the immunoregulatory pathways, such as Foxp3, IL-2 and its receptors, CD28, and TGFβ1 and its receptors may play critical roles in the onset of T1DM. Furthermore, the links between the immunoregulatory genes are likely underestimated in T1DM since, for example, Foxp3 loss-of-function mutations in humans cause the genetic disease immunodysregulation, polyendocrinopathy, enteropathy, X-linked syndrome (IPEX), which is a severe, typically fatal disease of mainly endocrine tissues that afflicts teenage boys (49, 50).

Single biologics, like non-FC-binding anti-CD3 antibodies to block TCRs, and thus “blinden” lymphocytes from “seeing” self-antigen, have been tested in T1DM pre-clinical animal models and human clinical trials of T1DM (17, 51). Non-FC binding anti-CD3 monoclonal antibodies (mAb) are antibodies that bind to CD3 to block the formation of the CD3/TCR/MHC complex but without triggering TCR signaling, since the antibody is not anchored on Fc receptors on accessory cells. Thus, the antibody prevents the T-cells from producing an adaptive T-cell-mediated immune response to the antigen (52). The use of non-FC-binding anti-CD3 mAb for the treatment of T1DM is an idea that has been tested for over 20 years, but unfortunately it does not cause sustainable T1DM disease remission in humans. A study of the effects of teplizumab (a non-FC-binding anti-CD3 mAb) on patients with T1DM indicated that patients who received treatment with the drug were able to attain some metabolic control (53). More recently, teplizumab treatment in patients with new-onset T1DM were shown to have a beneficial effect on preserving the C-peptide, which is an indicator of spliced insulin and, therefore, of its recent production. The authors noted that individuals who were responders to treatments had lower HbA1c, lower insulin use, and lower Th1-like cells prior to treatment (54). However, in a more recent phase III study of a similar drug Otelixizumab, treatment with a low-dose non-FC-binding anti-CD3 mAb was ineffective at preserving the C-peptide levels with no significant difference from the controls. The authors speculated that the failure of the treatment may have been because the dose was too low to achieve a measurable response (55). Overall, while some of these studies indicated that treatment with non-FC-binding anti-CD3 mAbs can display some efficacy on certain patient substrata, the failure of these TCR-blocking antibodies in the majority of patients place the future development of single-component TCR-blocking treatments into serious question.

CD28 signaling has also been targeted for immune-related diseases, whether boosting it for cancer immunotherapy, or attenuating it for autoimmune therapy (17). CD28 is highly expressed on T cells, and is bound by CD80 and CD86 on APCs. In addition, CTLA-4 is thought to compete with CD28 for binding to CD80 and CD86, thereby attenuating lymphocyte activation. Indeed, biologics designed around CTLA-4, such as CTLA-4 fused to FcR (brand name Belatacept) have been used successfully to treat a variety of autoimmune diseases and in organ transplantation (56) (Figure 4), and show some efficacy in T1DM animal models and clinical trials. More recently, the effects of CTLA-4 have been suggested to reside on Tregs. Nevertheless, since targeting signal 2 does not result in potentiation of effector and memory Treg responses, it is unclear if this approach alone can ultimately prove fruitful in T1DM immunotherapy to enable sustained islet regeneration or transplantation.

### Targeting the Growth Factor “Signal 3”

Therapy that seeks to manipulate the third signal (IL-2 and related cytokines), generally aims to selectively boost Tregs without setting off effector CD8 and NK cells. However, while this sounds good in principle, it has proven challenging in practice (57) since high doses of IL-2, while inducing the most Treg function, also induces/expands CD8 T cells and NK cells. Conversely, low-dose IL-2 therapy (58) may selectively enhance Tregs over CD8 T cells, but the dose may be very low as to be insignificant overall. IL-2 plays a critical role in the growth and expansion of T cells, reacting on the IL-2R. As such, the protein is currently being explored for both the treatment of cancer at higher doses and for autoimmunity at lower doses (57, 58). The high dose is recommended for its ability to expand effector T cells to increases the likelihood of lymphocyte infiltration of tumors (59). The overall success of the treatment was positive and suggests that for similar treatments, maximal tolerable doses of IL-2 should be administered for treatment in the interest of the greatest clinical outcome (60). In the case of autoimmunity, the utilization of low-dose IL-2 holds promise as a future treatment due to its ability to assist with the expansion of Tregs. This is made possible by the high affinity IL-2R (CD25) present on Tregs and lacking on T effectors (Teffs) (61). A recent study has suggested that the development of T1DM in NOD mice is in part due to Treg dysfunction caused by defective IL-2 production. The results of the study indicate the presence of an imbalance between Teffs and Tregs in inflamed islets, suggesting the Tregs present are insufficient to inhibit the autoreactive Teffs. The group found that treatment with low-dose IL-2 injections assisted in the prevention of T1DM development (62). Despite the usefulness of low-dose IL-2 in the production of Tregs, the presence of IL-2 similarly induces the expansion of Teffs as well (albeit less than high dose IL-2). To help combat this issue, a recent start-up company, Delinia created a specialized IL-2 mutein Fc fusion protein that specifically targets the receptors of Tregs thus avoiding induction of Teff proliferation (63). It is expected that this new modified IL-2 protein will be used in the future for Treg expansion studies.

### Targeting the T Cell Subset Polarization “Signal 4”

The third signal required to make *de novo* Tregs, TGFβ1, is a critical differentiation factor for expression of the Treg master transcription factor Foxp3. However, TGFβ1 can additionally lead to the differentiation of Th17 T cells if IL-6 is also present (64). Furthermore, TGFβ1 (when used alone) may exert side effects including cancer and fibrosis. As such, this serves as a cautionary warning for the modulation of proteins present when attempting to differentiate Tregs. Instead, multiple factors must be adequately considered when working to differentiate Tregs for immunotherapy, and considering that there may be multiple Treg subsets co-expressing Foxp3 and either T-bet, Gata3, or RORγt (33, 65), functional evaluation of the biologics or adoptively transferred cells remains decisive (66).

Various groups have considered different paths to induce naïve T cells toward the Treg phenotype. TGFβ1 has been implicated in the expansion of Tregs *in vivo*, including experimental autoimmune encephalomyelitis (EAE). During macrophage breakdown of the dying cells, TGFβ1 was released helping to stimulate the differentiation of autoantigen-specific Tregs (67). More recently, biodegradable particles loaded with autoantigen and TGFβ1 have been shown to improve EAE *via* Tregs (68). In a different approach, parasitic worms were found to secrete a TGFβ1-mimicing protein that amplified Tregs *in vivo* (69), raising the possibility that this can be developed as a biologic medication for autoimmune diseases and transplantation. However, the utilization of such biologics may face difficulty obtaining FDA approval due to the nature of the treatment. Regardless, methods including TGFβ1 stimulation show promise as a potential treatment avenue for both T1DM and several other autoimmune diseases.

### Adoptive Transfer of Regulatory Immune Cells

To address some of the challenges by using isolated biologics, cellular therapy aims to restore diseased tissues by transplanting tissue grown in the laboratory (70–73), or even better, by inducing and controlling tissue healing *in vivo* (74, 75). Since T1DM patients frequently have low Treg levels or impaired function of Tregs (72), treatments using adoptive transfer of Tregs to restore immune balance in T1DM patients have entered clinical trials, and thus far have been found to be generally safe (70, 73). Future phase II efficacy clinical trials will be a critical test of adoptive Treg therapy for autoimmunity and transplantation. Finally, there are recent attempts to bypass harvesting T cells and growing them in the laboratory by inducing or expanding Tregs *in vivo via* the utilization combined biologics, artificial APCs, or other approaches. These are emerging as novel “inverse vaccine” approaches to treat infections, cancer, or autoimmunity depending on the formulation (73, 75–78).

## Regenerative Medicine and Stem Cells

Stem cells have been broadly classified into either embryonic stem cells (ESC) or adult stem cells (ASC) (79). ESC are derived from the inner cell mass, are pluripotent, and give rise to three germ layers: ectoderm, mesoderm, and endoderm. Tissue-residing ASC play critical roles in supporting their local parenchymal tissue (80). These include mature muscle fibers located beneath the basal lamina, epidermal stem cells, located near the bulge region of hair follicles, and intestinal stem cells located near the bottom of intestinal crypts (79). ASC are known as tissue-specific stem cells because they can only produce a limited set of cells that belong to a particular progeny of cells, such as skin, muscle, intestine, blood, and nervous system. The most widely characterized multipotent stem cell populations are hematopoietic stem cells isolated from bone marrow (81, 82). In addition, multiple potent marrow stromal cells (MSC) can also be isolated from bone marrow to give rise to several mesenchymal lineages (83). However, there are still various tissue populations that lack tissue-specific markers that make isolating these stem cells ambiguous, and the connection of MSC to immunomodulation remains tenuous (84–88).

### Pancreatic Organogenesis and Stem Cells

There has been much skepticism and debate over the markers of liver and pancreatic stem cells (89–92), though cell lineage-tracing methods are now starting to provide unambiguous information on the origin of tissue-resident stem cells and their progenitors (79). Both embryonic and ASC present their own challenges; ASC are rare to find and extract as pure populations for *in vitro* growth and ESC are easier to grow into unspecialized cells, however, researchers are still struggling to come up with protocols that reliably differentiate these cells into specific cell types. Stem cells are also not always easy to culture *in vitro* and are highly sensitive to contamination or changing conditions (93).

Another issue faced in translation to clinical application concerns the fate of transplanted stem cells (94), in particular immunological rejection or the propensity to form tumors upon transplantation (benign teratomas, but also teratocarcinomas) (95). Transplanted stem cells may not work in conjunction with the individual’s own tissue, but may be rejected by the recipient’s own immune system, and may overgrow into cancer cells (93). There remain ethical concerns while dealing with either ESC or ASC (96). In a landmark discovery, Yamanaka and colleagues at Kyoto University in Japan in 2006 discovered methods to “reprogram” ASC back into a stem cell-like pluripotent state (97). These induced pluripotent stem cells (iPSC) represent a major breakthrough that paved the way for the use of iPSCs becoming a preferred stem cell source over ESC due to ethical concerns and risks of immune rejection (due to tissue type mismatching) in the latter.

### Induced Pluripotent Stem Cells

Induced pluripotent stem cells can bypass the need for an embryo to obtain ESC and can be derived in a patient-match manner. While iPSC have been regarded as a breakthrough to reduce concern of immune rejection, recent studies have indicated that iPSC-derived cells can be immunogenic and may not fully bypass immune responses (98). Thus, there still remains concern over the safety and genomic instability associated with iPSCs. Mouse embryonic or adult fibroblasts were the initial cell type used to produce pluripotent stem cells by introduction of four transcription factors: Sox2, Oct3/4, Klf4, and c-Myc, while maintaining ES cell culture conditions (97). Pluripotent stem cells such as iPSC and ES cells contribute largely to the field of regenerative medicine because they can be used to treat a variety of diseases including T1DM.

Induced pluripotent stem cells have been used to generate insulin-producing β-cells derived from human pluripotent stem cells, though the generated β-cells lack all of the functional characteristics of bonafide β-cells (99). Glucose-sensing insulin-secreting β-cells were derived in culture from two sources: human ESC and human iPSC. In order to identify insulin-producing cells within populations derived *in vitro*, two markers, such as NKX6-1 or PDX1 should be expressed. Functionally the stem cell-derived β-like cells should resemble islets of Langerhans, namely the cells should respond to high, but not low, glucose stimulation *in vitro*, along with the typical cellular responses involving calcium signaling and culminating in exocytosis of insulin granules. In an important 2014 study, human ESC were used to differentiate into PDX1/NKX6-1 positive cells (100, 101). Despite iPSCs having the markers indicating cell maturation comparable to bonafide β-cells, the stage 7 insulin-positive cells are functionally immature *in vitro*. This is because a mixed population of islet cells is observed due to the observation of insulin secretion decreasing at higher glucose concentrations compared to low glucose stimulation concentrations. This further demonstrates the inability to reach the full insulin-positive cell maturation stage. Both the proteomic analysis and physiological analysis demonstrated that insulin-positive cells were immature and required more factors to allow for complete maturation (102). Nevertheless, when these insulin-positive cells were transplanted into immunodeficient mice, they were able to mature further and demonstrated successful signs of curing diabetes in these mice. Therefore, this suggests that *in vivo* factors may be required to promote maturation of insulin-positive cells derived from iPSCs and that there are still factors missing from current iPSC differentiation protocols (101).

Another recent important study was conducted at Harvard by the Melton laboratory. This study first differentiated ESC into islet-like clusters and pancreatic progenitor cells expressing PDX1/NKX6-1 (103). These cells were then transplanted into mice and after 3–4 months, the transplanted cells demonstrated β-cells function. Upon culturing the cells *in vitro*, most SC-β-cells can be found within NKX6-1/C-peptide monohormonal population. Using flow cytometry, about one-third of the differentiated cells were shown to express NKX6-1/C-peptide, with a similar profile to cadaver islets. The remaining cells within the culture population of SC-beta clusters are either endocrine cells that express the GCG or SST hormones or PDX1 pancreatic progenitors that have yet to fully differentiate into mature endocrine cells. However, one issue that previous differentiation protocols have faced is the inability of the derived β-cells to resemble adult INS β-cells. Gene microarray analysis has shown that derived β-cells resemble fetal beta-cells rather than functional adult human β-cells. Nevertheless, this study demonstrated that SC-β-cells derived *in vitro* are comparably more similar to adult human β-cells than fetal β-cells even though there are still slight differences in gene expression. It has been speculated that the slight differences in gene expression could be due to the culture media or factors added to the cells that cause a shift in primary β-cells. Regardless of the expressed genes, these cells derived *in vitro* were capable of secreting insulin in response to glucose stimulation (102).

Another example illustrates the struggle of obtaining an appropriate yield of insulin-positive cells derived from human iPSC, and the need to correct any defective genes in patient iPSC before transplanting the corrected iPSC back to the patient. Fortunately, the recent breakthrough in genetic engineering, CRISPR-Cas methodology, promises to enable efficient gene correction in cells *in vitro* and potentially even *in vivo*, including pancreatic islet regeneration (104, 105). As this technology matures, it is expected to be the main approach for gene correction when mutations and the cell types they impact are known (106).

Finally, culturing iPSC and ES and subjecting them to differentiation protocols also requires practical skills and methods. For example, dissociation of human ESC can lead to increased vulnerability to apoptosis due to cell detachment and dissociation. To circumvent this problem, Y-27632 was tested as an inhibitor of p160-Rho-associated coiled-coil kinase (ROCK) in order to inhibit apoptosis (107). hESC were plated on mouse embryonic fibroblast feeder layer, pre-treated for 1-h with 10 μM Y-27632 in media, and were completely dissociated. Up to 30 passages with low-density plating, hES cells in the presence of 10 μM Y-27632 in media retained their expression profile of undifferentiated markers (Oct3/4 and E-cadherin) (107). These cells were then grafted into immunodeficient mice, which caused the formation of teratomas. Upon the use of a second ROCK inhibitor (10 μM Fasudil/HA1077), colony formation was enhanced for undifferentiated hESC. The two different ROCK inhibitors maintained similar colony formation. When hESC were treated with Y-27632, there was an increased cell growth and the formation of colonies when cultured with MEF cells (107).

### MSC as Stromal Cells That Support Islet Integration

As potent stromal and stem cells, MSC represent a new approach to facilitate acceptance and integration of biomedical implants and stem cell and organ transplants (84–88). MSC are rare but widely distributed stromal stem cells that play crucial roles in tissue repair and regeneration (108, 109). They hold great promise in regenerative medicine for their differentiation to adipocytes, chondrocytes, and osteoblasts to make cartilage and bones to replace damaged tissues (85, 108). In addition, MSC have also been reported to modulate immune responses across allogeneic barriers. Endogenous host MSC can be recruited from local tissue or bone marrow to sites of injury, such as ischemic heart, stroke, other wounds, as well as cancer (85, 108–113), and interestingly, to sites of pancreatic islet transplants (114, 115). MSC express specific antigen markers on their surface, such as CD73, CD105, and CD90 which can be used in flow cytometry to characterize MSC (116). Thus, MSC are under intense investigation for incurable and progressive autoimmune and inflammatory diseases, including for the protection of pancreatic islet cells in T1DM (108, 110, 112, 117, 118). MSC can currently be isolated and expanded from umbilical cord blood and umbilical cord matrix (116). Unlike current broad-spectrum immunosuppressive drugs mentioned above, MSC are versatile complex adaptive systems that respond to a variety of injury and inflammatory cues to secrete factors that generally reduce immune responses. These MSC-secreted factors include anti-inflammatory cytokines, such as TGFβ and cytokine signaling blockers (e.g., IL1-Ra, sTNF-R) (108, 118), prostaglandin E_2_ (PGE_2_) (119), TSG-6 (120), and local metabolic depletion *via*, e.g., indoleamine 2,3-dioxygenase (112).

Marrow stromal cells can be used from low passages in culture media, or can be additionally cultured under conditions that mimic tissue trauma, such as low growth factors, low oxygen, high levels of inflammatory cytokines (IFNγ, TNFα, and others), self-antigens, and damage-associated molecular patterns (DAMPs) prepared either as crude cell lysates or as purified components to exert their immunoregulatory functions (85, 108, 110, 112). The concept of sterile DAMPs as reporters or markers of certain types of disturbances of tissue homeostasis was introduced to extend the paradigm of self–nonself immune system discrimination in cases where such distinction would be difficult, yet the immune system would be alerted to tissue damage, resulting from cellular destruction or disruption of normal physiological functions by viable pathogens or major trauma (121–126). This tolerogenic property of DAMPs is being tested to induce immunological tolerance in autoimmune and inflammatory conditions (122, 124, 127, 128).

Marrow stromal cells have been used as a therapeutic potential in treating diabetes due to their regenerative abilities, ease of isolation, and low immunogenicity. MSC administered to 10 newly diagnosed T1DM patients resulted in promising C-peptide values (116). However, there was no difference observed for HbA1c or insulin dose when comparing control and MSC groups during phase I trial. Additionally, this therapy was also tested for T2DM in China. The study revealed the safety of the transplant and after 6 months, the T1DM biomarker HbALc began to improve along with higher C-peptide levels and insulin release (116). However, although MSC may prove to be a promising method to treat patients with diabetes, there is still no widely standardized procedure or protocol for their isolation, characterization, and therapeutic potency testing, though the field is moving in that direction (80, 116, 129).

## Biomedical Engineering for Islet Protection and Vascularization

**Figure 5:**
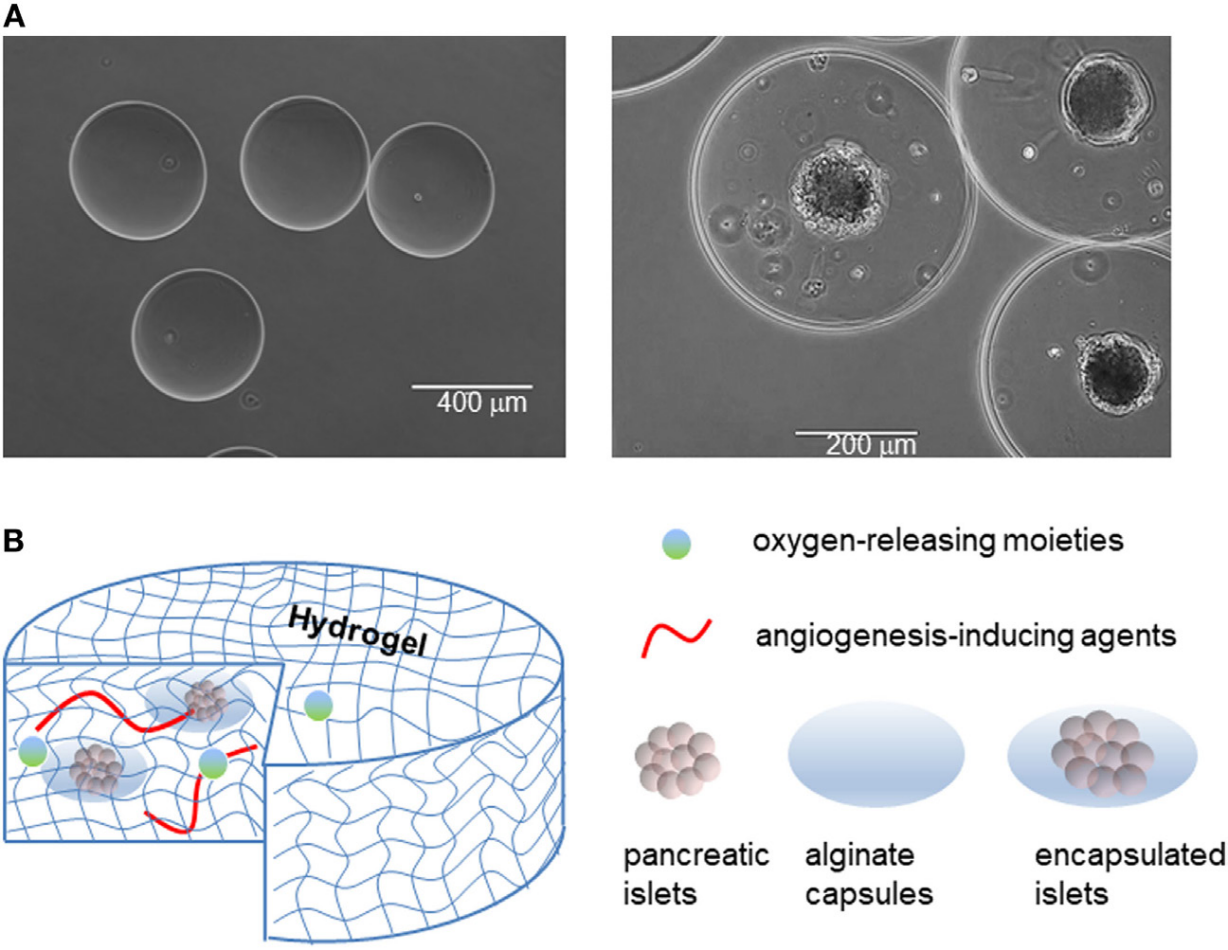
Islet encapsulation and their integration in medical devices for implantation in patient. **(A)** Empty alginate capsules (left panel), and alginate capsules containing islets (right panel). **(B)** Examples of devices to contain naked or encapsulated islets and features that enhance their integration with the body.

Islet transplantation is a possible method of treatment for T1DM first introduced by the Edmonton Protocol (130). The method involves obtaining pancreatic islet from cadaveric donors and then transplanting them into T1DM patients *via* the portal vein, which is much less cumbersome than the transplantation of a whole human pancreas. One of the hallmarks of the Edmonton Protocol during its creation was the utilization of immunosuppressants without glucocorticoids to prevent graft rejection (130). This method was tested relatively recently in a University of Chicago study of nine diabetic patients. Despite the dropping out of five subjects, the remaining four patients stayed insulin-free over the course of 5 years, receiving three islet infusions over that time (131). The successful repetition of this method suggests that islet transplantation hold promise as a future treatment for diabetes. However, issues still remain in the fact that few patients remain insulin free beyond 4 years post-transplantation. Other issues include a lack in availability of human islet donors, lack of facilities and proper training for the procedure, lingering side effects of immunosuppressants, and reversal of treatment over time (132). In order to circumvent the lack of human islet donors, effort has been made in trying to expand islet cellular masses *in vitro*. However, one caveat with culturing islets is that they decline in insulin production. Pig islets are another potential source for islets; however, two main concerns have limited the use of pig islets as a xenogeneic source for human transplantation. One concern is that humans express antibodies against a galactose residue present in most pig cells that could result in an immunological response against pig islet transplants. Second, pig cells contain retroviruses that may infect their human host. A third alternative for an islet resource is the potential use of insulin-producing cells, differentiated from ES cells or iPSC cells. Regardless of the source for islet transplants, there’s still the problem of the autoimmune response in T1DM patient that eventually leads to islet graft rejection. Therefore, it is highly important to address the need to bypass the immune response triggered by the transplantation of islets. For this reason, there has been exploration in using a semi-permeable membrane to shield transplanted cells from the immune system, prompting growth in the field of islet encapsulation (Figure 5A).

### Islet Encapsulation by Semi-Permeable Biomaterials

Islet encapsulation by biomaterials that allow diffusion of nutrients/waste, oxygen, and insulin but prevent infiltration by immune cells and antibodies has been explored as a candidate permanent treatment of T1DM for over 40 years (133–136). In practice, the method involves encapsulating islets in an immunoprivileged material protecting them from the adaptive immune system, while still enabling insulin to exit the capsule (137, 138). Through encapsulation, both graft rejection (due to allo/xeno-geneic and hESC transplant) and autoimmune attacks can be prevented (Figure 5).

Among the possible materials, sodium alginate, a polysaccharide derived from algae, is often used as the protective barrier to encase islets while allowing nutrient and waste exchange (133). The polysaccharide is first dissolved in water to produce the desired concentration, and is next polymerized into capsules using divalent cations, namely calcium (Ca^2+^) or barium (Ba^2+^) cations (139). Due to the difference in molecular size, the two different cations produce distinct alginate conformations, resulting in variations in capsule structural stability (139). However, sodium alginate capsules are prone to swelling when placed in hypotonic solution due to the imbalance in osmolarity, as we have observed in our experience. There is still much improvement needed for islet encapsulation engineering in order to optimize islet longevity. This includes parameters, such as co-encapsulation, protection against hypoxic stress, prevascularization, and good manufacturing practices (135). Furthermore, despite successes with encapsulated islet transplantation, it is necessary to continue studying optimal alginate composition as certain mixtures of alginate have been shown to stimulate fibrotic responses eventually leading to nullification of graft effectiveness (140).

During co-encapsulation, other molecules can be added to the capsule in order to enhance the performance of encapsulated islets (Figure 5B). An example of this is using a local immunosuppressive delivery molecule, such as dexamethasone, a corticosteroid, that can improve survival of encapsulated islets in recipients. Although islets encapsulated in an alginate hydrogel can protect islets from immune cells and antibodies, the alginate hydrogel cannot protect islets from smaller proinflammatory cytokines and other mediators released by immune cells. One way to overcome this problem is by using a silicon nanopore membrane after islet encapsulation in the thicker alginate capsule in order to further protect islets against cytokines, improve islet viability, and allow for continual appropriate insulin secretion in response to glucose levels (141). Alginate technologies are now beginning to incorporate aspects of immunomodulation as well, as illustrated in a macroporous alginate scaffold bearing TGFβ1 tethered throughout the structure. The device was able to harbor islets, enable vascularization, and produce an immunoregulatory microenvironment (142). Knowing that immune response generally initiates from the implants surface, other approaches to eliminate the immune response against the implanted biomaterials is to take advantage of surface chemistry (143). All the current approaches, however, could not completely resolve the immune response against implanted biomaterials. In addition, the outcomes of an implant biomaterial without host cells/proteins around it could create issues for long-term biomaterials integration. In this context, it seems that rather than attempting to eliminate the immune infiltration, biomaterials could be engineered to negotiate with the infiltrated cells and reprogram/skew them toward anti-inflammatory phenotypes. This could not only eliminate the side effects of immune infiltration but also aids the biointegration of the device over extended periods of time.

### Protection Against Hypoxic Stress

Another major issue that arises with islet encapsulation is avoiding oxygen deprivation leading to necrosis (144). Islets are highly sensitive cells, requiring a substantial amount of oxygen to function and survive. In order to accommodate this need, researchers have succeeded in transplanting islets into the portal vein (145) or pre-vascularizing transplant devices (135). To further improve oxygen supply to encapsulated islets, the angiogenic fibroblast growth factor 1 (FGF-1), was added to capsules to allow for the release of *in vitro* FGF-1 (146). In another study, solid peroxide in polydimethylsiloxane was encapsulated in addition to encapsulated islets in order to increase the amount of oxygen released in the region where encapsulated islets were placed (147). To further prevent hypoxic stress on implanted encapsulated islets, a vascularized matrix or scaffold can be implanted first in order to increase angiogenesis before the introduction of β-cells or encapsulated β-cells/islets. Using this method, islets implanted subcutaneously were able to restore normoglycemia compared to islets transplanted without prevascularization (134). However, challenges regarding prevascularization remain such as distribution of transplanted cells within the graft in order to appropriate the correct amount of space for islets and ensure that the integrity of the extracellular matrix remains uncompromised. One major challenge that bioengineers face with the engineering of microvesicles, is the fabrication and induction of microvesicles to include not only small single tubes of small vessels, but also a network of a vascular bed with an appropriate surface-area-to-volume ratio for the delivery of nutrients (148).

### Medical Devices to Integrate Encapsulated Islets

One way to improve both graft tolerance and oxygenation of transplanted islets is to use encapsulation devices (sometimes also referred to as macroencapsulation devices) containing hundreds to thousands of encapsulated islets along with some methods to improve gas and nutrient flow (149). Key functions of the device include capacity for insulin secretion in response to blood glucose levels, maintenance of cell viability, and improved vascularization to avoid hypoxic stress. Such device would be more responsive than insulin pumps because of the in-built natural feedback loop of islets in regulating insulin secretion.

Recently, the company Viacyte developed a device, Encaptra, hosting a thin membrane that is intended to protect transplanted β-cells from interacting with the body’s own immune cells, while allowing for the movement of nutrients and oxygen through the device (137, 138). The idea is that the membranes are capable of excluding proinflammatory cytokines from flowing into the device, while allowing sufficient glucose and insulin exchange. Generally, the sizes of these porous structures should remain in nanoscale. Recent works have shown that these types of devices could integrate within the host body with low foreign body response, and also prevent the priming of T cells toward antigen-specific phenotype (150). In August 2014, the device entered a phase I/II clinical trial with stem cells differentiated to various degrees toward pancreatic islet cells. Upon implantation of the device housing pancreatic progenitor cells, the expectation is that cells will more fully mature into human pancreatic cells (including β-cells) that can respond to a patient’s post-prandial high glucose levels in the blood. It is anticipated that growth of progenitor cells will assist by stimulating vascularization of the device to assist in mitigating issues with hypoxia. This trial is currently active, and primary outcomes are expected to appear after 2021, however, long-term studies in term of integration of the device into the body are required.

Another company, Beta-O2, developed the device Beta-Air in order to provide exogenous oxygen to encapsulated islets to meet their demand for high oxygen concentration for survival, basal metabolism, and active metabolism during insulin synthesis and insulin granule secretion (137, 138). The device is shaped like a disk and consists of two components: an islet module containing the encapsulated islet cells within an alginate hydrogel and a gas chamber that provides oxygen to those encapsulated islets. Implantation of the device is done subcutaneously, and the access ports are then placed to allow for daily oxygen refills. Islets will receive oxygen by passive diffusion of the oxygen from the gas chamber to the islet encapsulation chamber. Though this method provides an alternative to daily insulin injections, the extension of an external port to the inside of a patient’s body is concerning for an increased risk of infection. Similar to the pitfall of other implantable devices, should damage occur to the device, or the islets decrease in functionality, the patient is likely to be relegated to surgery to either change the device or refill it with islets.

In another approach seeking to optimize the implantation of encapsulation devices, the subcutaneous region for implantation is prevascularized before transplanting the islets. By enhancing vascularization before transplanting the encapsulated islets first, the increased microvascularization should increase islet cell survival of the encapsulated islets due to their high demand for oxygen (135). Examples of this approach include the Theracyte device and Sernova Cell Pouch. The Theracyte device was able to protect encapsulated pancreatic islets from allograft rejection in recipients that were nonimmunized, in an allogeneic rat model (diabetic Wistar-Furth) with Lewis rats as islet donors (151). Rats that received transplanted islets into the Theracyte device had a much higher transplant survival compared to rats receiving islets not held within the device. The Sernova’s Cell Pouch System is a medical device implanted to create a hospitable microenvironment for transplanted cells, with a pre-clinical study demonstrating the reversal of induced diabetes in a syngeneic mouse model, where islets were deposited into the subcutaneous device (152). Recipients that harbored the islets in the device were able to maintain normoglycemia. After the graft was analyzed, the islets stained positive for microvessels, insulin, and glucagon, indicating successful transplantation, survival, and function compared to failed engraftment in recipients receiving islets that were not contained in the device (152). A phase I/II clinical study is being conducted using the Sernova Cell pouch in diabetic patients.

### Challenges in Large-Scale Manufacturing and Compliance With Clinical Good Manufacturing Practice (cGMP) Standards

Engineering of encapsulation material for islet transplantation requires that cGMP standards can be used. This includes the choice of the material, purification method, shape of the device used, and the quality of the islets that are encapsulated. While alginate technologies hold promise, they have various challenges, such as swelling, endotoxicity of alginate, and oxygen starvation. Commercial alginates used for encapsulation technologies have been found to contain pathogen-associated molecular patterns, such as lipopolysaccharides (LPS or endotoxin), peptidoglycan, lipoteichoic acid, and flagellin. Other toxins that have been found include various other proteins and polyphenols (153). As a result, this can trigger the immune system against the encapsulated device graft and increased fibrotic overgrowth which prevents nutrient and waste diffusion within the site of the graft (135). The use of endotoxin-free substrates promotes cell survival, growth, and maturation (154), suggesting the necessity of using ultra-pure low viscous mannuronate alginate for improved islet transplantation outcomes.

## Conclusion and Future Directions

Diverse therapeutic approaches are in the pipeline for T1DM, ranging from immunomodulation and transplantation to a combination of the two or even the possibility to induce regulatory immune cells and differentiated islet cells *in vivo*. Biologics like antibodies and cytokines have been used clinically to tolerize the immune system with improved performance compared to the traditional small molecule drugs (steroids). Cellular therapy is a more recent approach to induce more specific immune suppression and stem cell-mediated regeneration. In this context, Tregs in particular have gained traction in recent years as a possible treatment for T1DM for their ability to suppress autoimmune responses without causing widespread immunosuppression. However, currently the greatest issue is producing effective and reproducible induction or expansion of Tregs.

Ultimately, the greatest issues surrounding the treatment of T1DM are ensuring that the quality of life for the patients increases and the treatments are viable for long periods of time. Although a patient may be treated with islet transplants and immunosuppressants, one must consider the possible deleterious side effects of the non-selective small molecule drugs. Likewise, if islet encapsulation is considered as a preferred method of treatment, issues such as nutrient/insulin exchange, protection from immune damage, hypoxia, and fibrosis of capsules must be overcome. One key issue with islet encapsulation is that the methods being used for the microencapsulation of islets can result in limitations in insulin diffusion due to the large capsule size (155). This may cause insulin secretion to be blunted, increase islet necrosis, and increase hypoxic risk. Therefore, it is important that implantation sites can be prepared prior to the transplantation of islet capsules, especially considering the relatively large volume of (encapsulated) islets or β-cells to be deposited. Other important factors include increased capsule and device stability, minimizing both engraftment volume and size, and reducing any immune responses (155).

With the rise of new technologies there remain several obstacles that must be overcome. This includes the fragility of transplant devices and possible necessity of islet “refills” to devices that must also be taken into consideration as well. Additionally, other approaches such as immunotherapy treatments using Tregs are becoming a rising approach in treating and potentially curing T1DM. Each treatment type has pitfalls and challenges that must be met; however, the greatest consideration beyond the efficacy of any treatment needs to be for the overall well-being and quality of life of the patient.

## Author Contributions

CK, TT, MA, MM, and EP contributed to the writing of this manuscript. JL reviewed the manuscript.

## Conflict of Interest Statement

The authors declare that the research was conducted in the absence of any commercial or financial relationships that could be construed as a potential conflict of interest.
